# Reproductive Outcomes in Cases of Subclinical Hypothyroidism and Thyroid Autoimmunity: A Narrative Review

**DOI:** 10.1055/s-0040-1714133

**Published:** 2020-12-21

**Authors:** Bruno Ramalho de Carvalho, Andrea Prestes Nácul, Cristina Laguna Benetti-Pinto, Ana Carolina Japur de Sá Rosa-e-Silva, José Maria Soares Júnior, Gustavo Arantes Rosa Maciel, Edmund Chada Baracat

**Affiliations:** 1 Hospital Sírio-Libanês, Brasília, Distrito Federal, Brazil; 2 Human Reproduction Unit, Hospital Fêmina, Grupo Hospitalar Conceição, Porto Alegre, Rio Grande do Sul, Brazil; 3 Department of Obstetrics and Gynecology, School of Medical Sciences, Universidade Estadual de Campinas, Campinas, São Paulo, Brazil; 4 Departamento of Gynecology and Obstetrics, Faculdade de Medicina de Ribeirão Preto, Universidade de São Paulo, Ribeirão Preto, São Paulo, Brazil; 5 Discipline of Gynecology, Department of Obstetrics and Gynecology, Hospital das Clínicas - HCFMUSP, Faculdade de Medina da Universidade de São Paulo, São Paulo, SP, Brazil

**Keywords:** thyroid diseases, thyroid function tests, thyroid hormones, hypothyroidism, autoimmunity, female infertility, doenças da tireoide, testes de função da tireoide, hormônios da tireoide, hipotireoidismo, autoimunidade, infertilidade feminina

## Abstract

Thyroid diseases are relatively common in women in the reproductive period. It is currently understood that clinically-evident thyroid disorders may impair ovulation and, consequently, fertility. However, to date it has not been proven that high serum levels of thyroid-stimulating hormone and/or positivity for antithyroid antibodies are associated to a reduction in fertility, mainly in the absence of altered thyroxine levels. The present comprehensive review aims to present current data on the association between subclinical hypothyroidism and/or thyroid autoimmunity and reproductive outcomes.

## Resumo

As doenças da tireoide são relativamente comuns em mulheres no período reprodutivo. Atualmente, entende-se que distúrbios da tireoide clinicamente evidentes podem prejudicar a ovulação e, consequentemente, a fertilidade. No entanto, não se provou até o presente que níveis séricos altos do hormônio estimulador da tireoide e/ou positividade para anticorpos antitireoidianos estão associados a uma redução na fertilidade, sobretudo na ausência de níveis alterados de tiroxina. Esta revisão narrativa tem como objetivo apresentar dados atuais sobre a associação entre hipotireoidismo subclínico e/ou autoimunidade tireoidiana e resultados reprodutivos.

## Introduction

The thyroid gland is responsible for regulating several mechanisms of human physiology, which include the reproductive function. Thyroid hormones are involved in the modulation of the hypothalamic-pituitary-gonadal axis and, despite the lack of consistent scientific evidence, it is currently understood that clinically evident thyroid disorders may impair ovulation and, consequently, fertility. 1 Thyroid diseases are relatively common in women in the reproductive period. A significant association between clinical thyroid disorders and abnormalities of the reproductive system has been largely confirmed: both primary hyperthyroidism and hypothyroidism have been documented to produce variable degrees of gonadal dysfunction. Nevertheless, the impact of subclinical thyroid dysfunction and/or thyroid autoimmunity (TAI) on fertility and reproductive outcomes is not consensual, although they may be related to infertility and the risk of spontaneous pregnancy loss. 1 2 As a matter of course, subclinical hypothyroidism (SCH) has been defined as a level of thyroid stimulating hormone (TSH) going over the upper threshold of 4.5 mIU/L to 5.0 mIU/L in the setting of a normal level of free thyroxine (fT4). 3 Nonetheless, the limits commonly vary among studies, and it has been suggested that the upper cutoff for TSH should be set at 2.5 mIU/L, based on the observation that 95% of asymptomatic people have that level or even lower levels of TSH. 4 Regarding thyroid diseases, in addition to idiopathic changes in function, situations resulting from the presence of autoantibodies are quite common, such as Hashimoto thyroiditis and Graves disease. Currently, at least three anti-thyroid antibodies can be evaluated in human serum: the thyroid globulin antibody (TGAb), the thyroid peroxidase antibody (TPOAb), and the thyrotropin receptor antibody (TRAb). However, presenting anti-thyroid antibodies is not sufficient to develop autoimmune thyroid disease, which pathophysiology is not yet fully understood. Thus, the clinical relevance of presenting positive antibodies without an established disease is still questionable, including the influence on fertility. 5 The present narrative review aims to present the current data on the association between SCH and/or TAI and reproductive outcomes. Our objective is to help clinicians decide the medical approach to women attempting to conceive and presenting those conditions, based on hierarchized evidence. However, once the levels of evidence do not provide a definitive judgment about the quality of the studies included nor they constitute a final recommendation, 6 clinicians may apply individualization as the main key for their critical appraisal in treatment decisions.

## Methods

Using the keywords *subclinical hypothyroidism* , *thyroid autoimmunity* , and *infertility* , we searched for clinical trials, controlled clinical trials, meta-analyses, and randomized controlled trials on the following databases: PubMed, the Cochrane Database of Systematic Reviews, the Cochrane Central Register of Controlled Trials, the Cochrane Gynecology and Fertility Group specialized register, and clinicaltrials.gov. No date or language restrictions were applied to the search. A total of 13 studies were primarily selected for this review. 7 8 9 10 11 12 13 14 15 16 17 18 19 The references of the selected studies were also checked, and seven more relevant articles were included. 20 21 22 23 24 25 26 The evidence was hierarchized according to the Oxford Centre for Evidence-based Medicine's 2011 Levels of Evidence 6 by the first author (BRC), and checked by the second author (APN); there were no discordances between them or between them and the other authors.

### Subclinical Hypothyroidism

Subclinical hypothyroidism is a condition in which the level of TSH is elevated, but the level of fT4 is normal. It represents an early, mild thyroid failure, and affects up to 10% of the adult population. 27 However, its clinical significance has not been consistently proven. Raber et al 7 followed 223 women for up to 5 years, and they observed lower conception rates among women who never achieved a basal TSH < 20 mUI/L with fT4 therapy, and, then, they suggested a negative effect of such findings on reproductive function (level 4). Moreover, the meta-analysis of two studies 21 showed a significant decrease in the rates of miscarriage (relative risk [RR]: 0.18; 95%; 95% confidence interval [95%CI]: 0.08–0.39; *p* < 0.01) and preterm delivery (RR: 0.41; 95%CI: 0.24–0.68; *p* = 0.0005) in women with SCH treated with levothyroxine (LT4) (level 1). However, contrary to those authors, preconception TSH ≥ 2.5 mIU/L was not associated to time to biochemical pregnancy (odds ratio [OR]: 1.09; 95%CI: 0.90–1.31), pregnancy loss (RR: 1.15; 95%CI: 0.86–1.54) or live births (RR: 1.01, 95%CI: 0.89–1.14) among 1,193 women with normal fT4 (0.7 ng/dL to 1.85 ng/dL) and a history of either one or two previous pregnancy losses, even if they were positive for anti-thyroid antibodies (TGAb ≥ 115 IU/mL and/or TPOAb ≥ 35 IU/mL), and after adjusting for age and body mass index. The authors also attempted to determine a TSH cut-off affecting the continuation of pregnancy, but an additional analysis of both TSH tertiles and continuous TSH did not result in differences between women with TSH ≥ 2.5 mIU/L and those with TSH < 2.5 mIU/L (level 2). 12 There are some studies evaluating the effect of LT4 in women with subclinical hypothyrodism undergoing assisted reproductive treatments, like in vitro fertilization (IVF), with conflicting results. 8 10 15 22 26 According to Kim et al, 10 women with subclinical hypothyroidism undergoing assisted reproductive techniques presented with similar clinical pregnancy rates when compared with controls, despite the significant differences in the number of good-quality embryos, implantation rates, and live-birth rates (RR: 1.8; 95%CI: 1.0–3.25; *p* = 0.05; and RR: 2.13; 95%CI: 1.07–4.21; *p* = 0.03 respectively). 10 However, the miscarriage rate was significantly lower in the LT4 group (no miscarriages *versus* 33,3% in the control group; *p* = 0.021) (level 2). In another trial, 8 LT4 or placebo were initiated one month before IVF and were maintained throughout pregnancy. The number of follicles punctured, mature oocytes, and the fertilization, pregnancy, and delivery rates were significantly higher in the treatment group. Moreover, the miscarriage rate was significantly lower in the intervention group (level 1). 8 In a cohort study by Cai et al, 15 270 women with SCH supplemented with LT4 before IVF were compared with 200 age-matched euthyroid women who underwent classical IVF or intracytoplasmic sperm injection (ICSI). In total, 176 out of 270 women completed all pregnancy visits of the study, and were included in the final analysis. In the SCH treated with LT4 and euthyroid groups of women who underwent IVF in the same period, the overall rates of clinical pregnancy (44.31% versus 38.36%; *p* = 0.251 respectively) and miscarriage (10.3% versus 10.7%, *p* = 0.39 respectively) were similar (level 3). 15 Moreover, another study 12 demonstrated that the treatment with LT4 lead to the same rates of clinical pregnancy, miscarriage, and live births, which were independent of TSH levels, in women with SCH. There were no differences between the groups regarding the total number of oocytes retrieved and good-quality embryos (level 2). 12 To enhance the challenge, two recent meta-analysis have shown that LT4 supplementation in women with SCH can significantly reduce the risk of miscarriage after assisted reproductive technologies (ARTs) in almost 50% (RR: 0.51; 95%CI: 0.32–0.82), but not the rate of preterm birth (RR: 1.13; 95%CI: 0.65–1.96). In women with TAI, LT4 supplementation reduced the risks of pregnancy loss (RR: 0.61; 95%CI: 0.39–0.96; *p* = 0.03) and preterm birth (RR: 0.49; 95%CI: 0.30–0.79; *p* = 0.003) in naturally-conceived pregnancies, but not in pregnancies achieved by ARTs (level 1). 17 These results support previous similarly-designed studies (level 1). 16 22 As a matter of fact, the last Cochrane review 23 concluded that evidence is not sufficient to support the recommendation of one prepregnancy or mid-pregnancy intervention over another, in cases of SCH (level 1). Given that their findings were only based on two trials with a moderate risk of bias, and that new trials have been published after that, the conclusion of a reduction in preterm birth and a trend toward reduced miscarriage with the use of LT4 therapy should be taken with caution when deciding to treat euthyroid women. More recently, a Cochrane systematic review 18 evaluated the LT4 treatment in subfertile women with SCH undergoing ARTs. Only in one study involving 64 women with both subclinical hypothyroidism and positive or negative TPOAb, LT4 replacement provided an improvement in the rate of live births (RR: 2.13; 95%CI: 1.07–4.21), with similar miscarriage rates (RR: 0.11; 95%CI: 0.01–1.98) (level 1). 18 Nevertheless, the authors could not draw clear conclusions due to the low to very low quality of the evidence reported.

Finally, according to the American Society for Reproductive Medicine, 3 evidence that SCH (defined as TSH > 2.5 mIU/L with a normal level of fT4) affects fertility or induces miscarriages is insufficient. In the absence of specific recommendations for women attempting pregnancy, there is a suggestion to use pregnancy thresholds to minimize the potential risks associated with SCH. The American Thyroid Association 2 has published recommendations on the thresholds; briefly, in the absence of TAI, LT4 replacement is recommended for women presenting with TSH > 10.0 mIU/L (strong recommendation, but based on low-quality evidence), and could be considered for those presenting with TSH ≥ 4.0 mIU/L and < 10.0 mIU/L (weak recommendation, also based on low-quality evidence).

### Thyroid Autoimmunity

Thyroid autoimmunity seems to be relatively common among women of reproductive age, and it might be associated with subfertility and adverse pregnancy outcomes, like miscarriage, recurrent miscarriage and preterm birth. Although not consensual, the literature suggests that the administration of LT4 can improve reproductive outcomes in women with normal thyroid function and positive thyroid autoantibodies (level 1). 9 20 28

First of all, the association between TAI and impaired fertility is still to be proven. In the study by Plowden et al, 12 TAI (TGAb ≥ 115 IU/mL and/or TPOAb ≥ 35 IU/mL) was examined in relation to time to biochemical pregnancy, pregnancy loss, and live birth among women with normal fT4 with one or two previous pregnancy losses. The authors did not find a significant delay in pregnancy (OR: 1.11; 95%CI: 0.88–1.40), higher risk of pregnancy loss (RR: 0.90; 95%CI: 0.61–1.33) or impaired live birth rate in women with circulating anti-thyroid antibodies (RR: 1.04; 95%CI: 0.90–1.20), even after adjusting for age and body mass index (level 2). 12

Addiotnally, according to van den Boogaard et al, 9 29 no association was found between TAI and the rates of clinical pregnancy after IVF in the meta-analysis of seven studies (OR: 0.71; 95%CI: 0.36–1.4). However, the same study found elevated odds for unexplained subfertility (OR: 1.47; 95%CI: 1.06–2.02; *p* = 0.02), miscarriage (OR: 3.73; 95%CI: 1.83–7.6; *p* = 0.0003), recurrent miscarriage (OR: 2.26; 95%CI: 1.46–73.5; *p* = 0.0003), and preterm delivery (OR: 1.93; 95%CI: 1.08–3.47; *p* = 0.03) among euthyroid women positive for thyroid autoantibodies (level 1). 9 29

In the same sense, the meta-analysis 20 of seven homogeneous cohort studies demonstrated a significant elevation in the odds of miscarriage among subfertile women presenting with thyroid autoantibodies (OR: 3.15; 95%CI: 2.23–4.44; *p* < 0.001), especially TPOAb, but such an association was not proven by analyzing the three eligible studies involving women with recurrent pregnancy loss. Moreover, the authors found a 2-fold increase in the odds of preterm birth in the presence of TAI (OR: 2.07; 95%CI: 1.17–3.68; *p* = 0.01), with a significant 52% reduction in the relative risk of miscarriage (RR: 0.48; 95%CI: 0.25- 0.92; *p* = 0.03) and a 69% reduction in the relative risk of preterm birth (RR: 0.31 95%CI: 0.11–0.9; *p* < 0.05) when LT4 was supplemented in women with thyroid autoantibodies (level 1). 20 Finally, in a recent meta-analysis, Dong et al 19 showed an association between TAI and recurrent pregnancy loss (OR: 1.94; 95%CI: 1.43–2,.4), but LT4 did not improve the pregnancy outcomes (level 1). 19

Despite the aforementioned findings, Vissenberg et al 21 could not demonstrate the benefits of treating euthyroid women with positive thyroid autoantibodies with LT4 (level 1). 21 In a preview cohort study, Raber et al 7 did not find a significant association between the presence of TPOAb and TGAb and pregnancy or abortion rates in infertile women with or without SCH followed-up for more than 5 years (level 4). In accordance to those results, Dhillon-Smith et al 25 could not find significant differences in the rates of live births after at least 34 weeks of pregnancy by using 50 µg of LT4 once a day, started before conception and continued throughout pregnancy, among euthyroid women with TPOAb with a history of miscarriage or infertility. There was also no significant effect of LT4 on other pregnancy or neonatal outcomes, including the incidence of miscarriage and preterm birth (level 1). 25

Another trial 14 evaluated the treatment with LT4 initiated between 2 and 4 weeks before the controlled ovarian hyperstimulation for IVF and continued through the end of pregnancy in women with normal thyroid function who tested positive for TPOAb. The LT4 treatment did not reduce rates of miscarriage or improved the rates of live births compared with the usual care (level 1). 14 A recent Cochrane Systematic Review 18 also showed no differences in miscarriage rates or live-birth rates with the LT4 treatment or placebo in a similar group of women undergoing ARTs (level 1).

Beside all the lack of evidence to support the use of LT4 in euthyroid women with positive antibodies, Bartáková et al 11 analyzed the reproductive outcomes of 258 women up to 47 months after an episode of spontaneous abortion in the first trimester, and 43% of them were “positive for thyroid disorders” (level 3). Despite the fact that they found a significantly lower rate of secondary infertility among women treated with LT4 when compared with the controls and untreated women (4.1% versus 10.9% versus 21.1% respectively), such a finding was not clear when they compared the controls to treated and untreated positive women together (10.9% versus 9.9% respectively). The authors concluded that screening for thyroid disorders in women after spontaneous abortion and treatment with LT4 is cost-saving and improves the subsequent pregnancy rate.

In the absence of specific recommendations for women attempting pregnancy, some defend the use of pregnancy thresholds to minimize the potential risks associated with TAI. According to the most recent American Thyroid Association (ATA) recommendations, 2 pregnant women presenting with TSH > 2.5 mIU/L should be regularly evaluated for TPOAb; briefly, for those TPOAb-positive, LT4 therapy is recommended if TSH ≥ 4.0 mIU/L (strong recommendation, based on moderate-quality evidence), and could be considered for those presenting with TSH > 2.5 mIU/L and < 4.0 mIU/L (weak recommendation, also based on moderate-quality evidence).

### Practical Aspects

In the absence of sufficiently consistent scientific evidence on the approach of thyroid function in women attempting to conceive, and considering the aforementioned findings, we propose the following practical aspects:

1. Healthy women actively attempting to conceive should not be evaluated for thyroid disorders (strong recommendation, evidence of moderate quality); 12
2. Infertile women should be evaluated for thyroid disorders 3
3. In infertile women presenting with TSH > 2.5 mIU/L, evaluate the TPOAb:
  - The LT4 therapy is recommended (strong recommendation, evidence of low to moderate quality) for women presenting:
    - TPOAb-positive, TSH ≥ 4.0 mIU/L;
    - TPOAb-negative, TSH > 10.0 mIU/L;
  - The LT4 therapy may be individually considered (weak recommendation, evidence of moderate quality) for women presenting:
    - TPOAb-positive, TSH > 2.5 mIU/L and < 4.0 mIU/L;
    - TPOAb-negative, TSH ≥ 4.0 mIU/L and < 10.0 mIU/L; 2
4. The LT4 therapy is not recommended (strong recommendation, evidence of high quality) for women presenting:
  - TSH ≤ 2.5 mIU/mL;
  - TPOAb-negative, TSH < 4.0 mIU/L. 2

### Laboratorial Pitfalls

Thyroid function tests (TFTs) are routinely ordered, but the evaluation and interpretation of the results may be difficult at times due to technical problems. The pitfalls in the hormonal evaluation can be preanalytical, analytical, and postanalytical. The preanalytical factors include age, pregnancy, use of medications (such as oral contraceptives and biotin), genetic mutations, systemic diseases, and critical illnesses. The analytical errors occur due to heterophile antibodies and macro-TSH. The postanalytical errors include wrong registration of the result by the laboratory, mistakes in the units of the parameter checked, and failure to identify the normal data. 28 30 Thus, before taking clinical decisions, it is important that the physician become aware of those challenges and repeat the test in case of doubt.

## Final Considerations

The decision to treat SCH, particularly in women attempting pregnancy and infertile women, remains controversial, since the current understanding of the effect of thyroid dysfunction and/or autoimmunity on reproductive outcomes is based largely on low quality evidence. For this reason, the findings on the reproductive influence of SCH and TAI should be considered with care. Also because of the lacking evidence, the treatment with LT4 should not be established as a routine for women with SCH or those positive for thyroid autoantibodies as isolated findings, even assuming that potential benefits may outweigh the potential risks. As a matter of fact, the use LT4 will certainly benefit pregnant women with clinical hypothyroidism, and is an accepted strategy for those with a combination of TAI and elevated TSH. The treatment may be extended with caution and informed consent when that combination is found in subfertile women or those attempting pregnancy, but future better-designed studies are expected to support strong recommendations. Finally, screening for thyroid dysfunctions may be considered reasonable in women who are attempting to conceive and in the initial stage of pregnancy, but this is not consensual. Regarding the treatment with LT4, it is well established only in cases of clinical hypothyroidism, but it should be accepted in the following situations: 1) SCH associated to infertility; 2) TAI with TSH ≥ 4.0 mIU/L; or 3) when TSH > 10.0 mIU/L. Therefore, it should not be a rule for subclinical conditions, especially in the absence of autoimmunity. In the case of an individualized treatment for those who are candidates for maternity, we suggest that the same guidelines provided for pregnancy should be followed.
